# Engineered Extracellular Vesicles as Programmable Immune Interfaces: Surface and Cargo Engineering for Cancer Immunotherapy and Tolerance

**DOI:** 10.3390/cells15131213

**Published:** 2026-07-03

**Authors:** Tomoyoshi Yamano, Rikinari Hanayama

**Affiliations:** 1 Department of Immunology, Graduate School of Medical Sciences, Kanazawa University, 13-1 Takara-machi, Kanazawa, Ishikawa 920-8640, Japan; 2 WPI Nano Life Science Institute (NanoLSI), Kanazawa University, Kakuma-machi, Kanazawa, Ishikawa 920-1192, Japan

**Keywords:** extracellular vesicles, EV engineering, immune regulation, surface display, cargo loading, cancer immunotherapy, MSC-EV, clinical translation

## Abstract

Extracellular vesicles (EVs) are membrane-enclosed nanoparticles that mediate intercellular communication in the immune system by transferring proteins, nucleic acids, and lipids. Their biocompatibility, nanoscale size, and capacity for cell-type-selective delivery have stimulated growing interest in engineering EVs as therapeutic platforms. In this review, we discuss recent advances in EV engineering for immune regulation, focusing on surface display, cellular targeting, and cargo loading strategies. A central concept is that engineered EVs should not be viewed simply as delivery vehicles, but as programmable immune interfaces. EVs can integrate antigen specificity, target-cell recognition, therapeutic cargo delivery, and defined immunostimulatory or tolerogenic signals within a single nanoscale particle. By combining these modular elements, engineered EVs can be designed to direct immune responses in a context-dependent manner. We examine how this principle is being applied to cancer immunotherapy, immune suppression, and antigen-specific tolerance induction, including antigen-presenting EVs, cytotoxic and RNA-loaded EVs, checkpoint-modulatory EVs, MSC-derived EVs, and engineered platforms for autoimmune and inflammatory diseases. We also discuss the clinical translation of engineered EV therapeutics, with emphasis on manufacturing, characterization, potency assays, biodistribution, safety, and regulatory challenges. Together, current advances suggest that programmable EV immune interfaces may provide a versatile foundation for next-generation cancer immunotherapy and antigen-specific immune regulation.

## 1. Introduction

Extracellular vesicles (EVs) are membrane-enclosed nanoparticles released by virtually all cell types into the extracellular space under both homeostatic and stimulated conditions. Rather than forming a single homogeneous entity, they span a spectrum of particles that differ in biogenesis, size, molecular composition, and biological activity. Because rigorous assignment of biogenesis is rarely achievable in practice, the MISEV2023 guidelines recommend an operational nomenclature based on isolation method, physical properties, and molecular markers rather than presumed cellular origin [1]. We adopt this operational framework throughout the review, while retaining terms such as “exosome,” DEX, or iExosomes where they are used for established preparations in the original literature.

In the immune system, EVs play multifaceted and often opposing roles. EVs released by immune cells, including dendritic cells, T cells, NK cells, and macrophages, can carry antigen–MHC complexes, co-stimulatory ligands, cytokines, and immunomodulatory RNAs that shape both innate and adaptive responses [2]. Conversely, tumor-derived EVs exploit the same biology to suppress anti-tumor immunity through surface molecules such as PD-L1, FasL, and TGF-β, as well as nucleic acid cargoes that impair dendritic cell maturation, expand regulatory T cells (Treg), and dampen NK and T cell activity [3]. The net immunological outcome of EV signaling is therefore determined by the cellular source, cargo, surface molecules, and identity of the recipient cell, each of which can, in principle, be rationally engineered.

**Figure 1:**
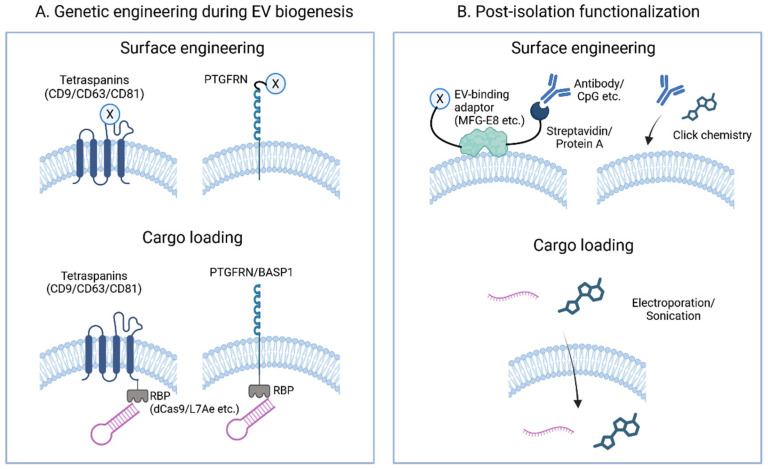
Strategies for engineering the surface and luminal cargo of extracellular vesicles. EV engineering approaches are organized by when the modification is introduced. (**A**) Genetic engineering during EV biogenesis. Functional moieties are genetically encoded in the producer cell and incorporated into EVs as they form. For surface engineering (**top**), tetraspanins (CD9, CD63, CD81) display a protein of interest (X) inserted into an extracellular loop, whereas single-pass scaffolds such as PTGFRN (and related scaffolds, e.g., Lamp2b and PDGFR-TM) present X on their ectodomain. For cargo loading (**bottom**), scaffolds are fused on their cytoplasmic side to RNA-binding proteins (RBPs; e.g., HuR or MS2 coat protein) or inducible protein-interaction domains (e.g., dCas9 or L7Ae) that recruit RNA or protein cargo into the EV lumen. (**B**) Post-isolation functionalization. Purified EVs are modified exogenously after isolation. For surface engineering (**top**), EV-binding adaptor proteins (e.g., MFG-E8) can anchor functional moieties to the EV membrane, and adaptor-based systems such as streptavidin or protein A can be used to attach biotinylated ligands, antibodies, CpG, and related cargoes. Bioorthogonal click chemistry provides an additional chemical strategy for covalent surface functionalization. For cargo loading (**bottom**), membrane-permeabilizing methods such as electroporation or sonication enable direct loading of nucleic acids or small-molecule drugs into the EV lumen. X denotes a displayed functional moiety, such as a targeting ligand, antigen, or immunomodulatory signal.

**Figure 2:**
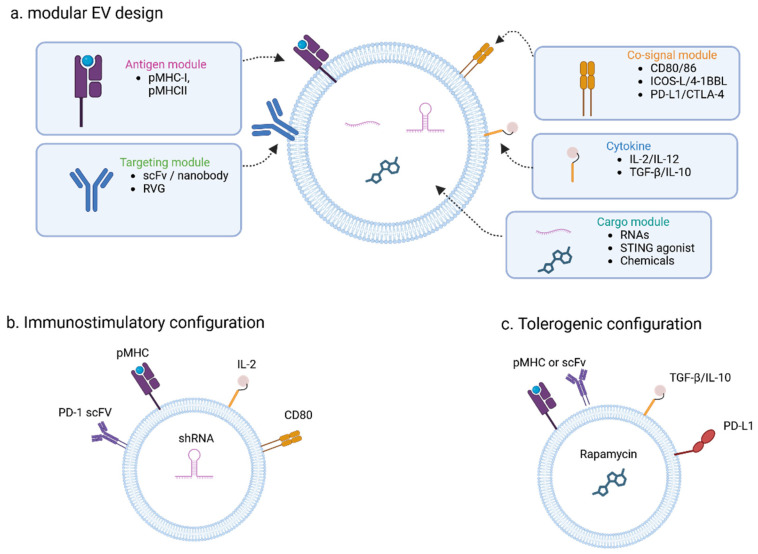
Engineered EVs as programmable immune interfaces: modular design and context-dependent configurations. (**a**) Modular EV design. Engineered EVs can be assembled from interchangeable functional modules that together define their immunological output. The antigen module presents peptide–MHC class I or II complexes (pMHC-I, pMHC-II); the targeting module confers cell-type selectivity through displayed ligands such as scFv or nanobody fragments or the rabies virus glycoprotein-derived peptide (RVG); the co-signal module delivers stimulatory or inhibitory signals via surface-displayed CD80/86, ICOS-L/4-1BBL, or PD-L1/CTLA-4; the cytokine module provides tethered cytokines such as IL-2/IL-12 or TGF-β/IL-10; and the cargo module carries luminal payloads including RNAs, STING agonists, or small-molecule drugs. (**b**) Immunostimulatory configuration. Combining an antigen (pMHC) with a stimulatory co-signal (CD80), a pro-inflammatory cytokine (IL-2), a checkpoint-blocking antibody (anti-PD-1), and a gene-silencing cargo (shRNA) yields an EV tailored to prime and amplify anti-tumor immunity. (**c**) Tolerogenic configuration. Combining an antigen or targeting ligand (pMHC or scFv) with an inhibitory ligand (PD-L1), a suppressive cytokine (TGF-β/IL-10), and an immunoregulatory cargo (rapamycin) yields an EV tailored to promote antigen-specific tolerance. The same modular platform can thus be reconfigured to drive opposing immune outcomes depending on the combination of loaded signals.

The natural biocompatibility of EVs, their nanoscale dimensions, their capacity for receptor-mediated uptake, and their potential to access selected biological compartments make them attractive therapeutic platforms [4]. Engineering approaches fall into two broad categories that also define the structure of this review: modification of the EV surface to confer targeting specificity or present immunological signals, and loading of the EV lumen or membrane with therapeutic cargoes (Figure 1). Over the past decade, systematic engineering of both surface and cargo has expanded EV functionality beyond passive transport, with applications in cancer immunotherapy, tolerance induction, and ex vivo cell engineering. Building on these advances, we frame engineered EVs not merely as delivery vehicles, but as programmable immune interfaces assembled from three modular components: a targeting element, a surface-signal module, and an intracellular cargo payload. This logic can be adapted to diverse recipient cells, including T cells, NK cells, macrophages, dendritic cells, and tumor cells (Table 1). By varying the combination of loaded signals, the same modular platform can be configured to either amplify or restrain immunity, yielding immunostimulatory and tolerogenic EVs from a common design framework (Figure 2).

However, this modular view also raises two practical requirements for EV engineering. First, the intended modules must be physically integrated on the same vesicle. Co-expression of multiple modules in producer cells does not necessarily guarantee their co-residence on individual EVs, because any given scaffold may decorate only a fraction of the total EV pool. Integrated multi-module designs therefore require single-vesicle validation rather than inference from bulk co-expression. Second, the programmed input must generate a functional output in the recipient cell. This makes it essential to distinguish productive intracellular delivery or signaling from mere cellular uptake when interpreting EV platforms.

The review is organized along these axes. We first describe EV surface display (Section 2) and cargo loading (Section 3), and then discuss therapeutic applications in cancer immunotherapy (Section 4), and immune modulation and tolerance induction (Section 5), before addressing clinical translation, manufacturing, and safety considerations (Section 6), and future directions (Section 7). We prioritized primary studies that report intentionally engineered EVs (surface display, defined cargo loading, or producer-cell modification) together with mechanistic, biodistribution, or clinical readouts. Naive or preconditioned MSC and stem-cell EVs and antigen-loaded dendritic cell EVs (Dex) are cited only as comparators, and broader EV biology is summarized through authoritative reviews.

## 2. Surface Engineering of Extracellular Vesicles

Displaying functional moieties on the EV outer membrane is a foundational strategy for conferring target specificity, enhancing cellular uptake, and co-presenting therapeutic signals. The scaffold used for surface display influences which EV populations are modified, how efficiently the displayed molecule is incorporated, and whether the modification occurs during EV biogenesis or after EV isolation. In this section, we summarize representative strategies for EV surface engineering, including genetically encoded display systems and post-isolation functionalization approaches. Figure 1 provides a comparative overview of representative surface display modules discussed in this review.

### 2.1. Tetraspanin-Based Scaffolds

Tetraspanins such as CD9, CD63, and CD81 are among the most widely used genetically encoded scaffolds for EV engineering. CD63 was initially used as a fluorescent fusion partner to track exosome biogenesis and trafficking [5], and was later developed as a scaffold for “exosome surface display” by inserting reporter proteins into its second extracellular loop [6]. This strategy demonstrated that tetraspanins can be used to present proteins on the outer surface of EVs. Tetraspanin-based scaffolds have been widely used to display targeting peptides [7], decoy receptors [8], antibody fragments [9], cytokines [10] and other functional proteins on EV surfaces. They have also been adapted for intraluminal cargo loading strategies, either by directly fusing cargo proteins to the cytoplasmic side of tetraspanins or by attaching RNA-binding proteins and inducible protein-interaction domains that recruit specific cargoes into EVs [11,12,13,14]. Thus, tetraspanins provide versatile genetically encoded scaffolds for both EV surface display and cargo enrichment. An important consideration, however, is that CD9, CD63, and CD81 are not uniformly distributed across all EVs [15,16]. Single-vesicle and EV-subtype analyses indicate that these tetraspanins mark only partially overlapping vesicle populations, with CD63 and CD81 in particular reported to be enriched on distinct EV subsets depending on producer cell type and biogenesis route [17]. Consequently, a given tetraspanin scaffold may decorate only a fraction of the total EV pool, and co-expression of multiple tetraspanin-based constructs does not guarantee that the separately displayed or loaded modules will reside on the same vesicle. When two or more functional modules are designed to function together on the same EV, their co-packaging into individual vesicles should be experimentally validated, rather than inferred from co-expression in producer cells.

### 2.2. Lamp2b-Based Scaffolds

Lysosomal-associated membrane protein 2b (Lamp2b) localizes to late endosomal and lysosomal compartments and can be incorporated into secreted small EVs, making it an attractive scaffold for N-terminal display of targeting peptides [18]. A landmark study by Alvarez-Erviti and colleagues showed that fusion of the rabies virus glycoprotein-derived RVG peptide to Lamp2b enabled intravenous EV-mediated delivery of siRNA to the mouse brain and knockdown of the neuronal target BACE1 [19]. This provided an early demonstration that Lamp2b can be used as an EV surface-display scaffold for tissue-targeting peptides. This strategy was extended by Tian and colleagues, who engineered immature dendritic cell-derived EVs to display the tumor-penetrating peptide iRGD and loaded them with doxorubicin [20]. iRGD-modified EVs showed enhanced tumor targeting and improved antitumor efficacy compared with non-targeted EV-doxorubicin formulations in preclinical tumor models. These studies established Lamp2b as a useful scaffold for directing EV biodistribution through peptide display. A key limitation of Lamp2b-based peptide display is that extracellular peptide fusions may be susceptible to proteolytic degradation during EV biogenesis, particularly in acidic endosomal compartments. Hung and Leonard addressed this issue by introducing N-linked glycosylation motifs near the peptide-Lamp2b fusion junction, which protected displayed peptides from proteolytic cleavage and increased the abundance of intact fusion proteins in secreted EVs [21]. Thus, glycoengineering can improve the stability of Lamp2b-displayed peptides and may be particularly useful when extracellular peptide degradation limits surface display.

### 2.3. MFG-E8/Lactadherin as a PS-Binding Anchor

Milk fat globule-EGF factor 8, also known as MFG-E8 or lactadherin, contains C1 and C2 domains that bind phosphatidylserine exposed on the outer leaflet of EV membranes [22]. Protein fusions incorporating MFG-E8 or its C1C2 domain can therefore be displayed on EVs either by expression in producer cells or by post-isolation decoration of purified EV preparations [23,24]. The latter approach enables modular surface functionalization without genetic modification of the original EV-producing cells, which is useful when producer-cell engineering is impractical or when the same EV preparation needs to be modified with different functional proteins. As a therapeutic example, Lyu and colleagues fused an anti-spike nanobody and IFN-β to MFG-E8 to decorate exosomes through phosphatidylserine binding [25]. These nanobody-IFN-β-conjugated exosomes inhibited spike-ACE2 interaction and reduced SARS-CoV-2 pseudovirus infection, illustrating how MFG-E8-based anchoring can confer both targeting and therapeutic effector functions on EVs. MFG-E8/C1C2-based anchoring has also been applied to analytical EV engineering, such as TurboID-EV, in which an MFG-E8-based construct was used to label proteins near internalized small EVs [26]. Because C1C2-based anchoring relies on phosphatidylserine exposure, its performance may vary depending on EV source, isolation method, membrane lipid composition, and the relative abundance of phosphatidylserine-positive vesicles.

### 2.4. PTGFRN-Based Scaffolds

Prostaglandin F2 receptor negative regulator (PTGFRN) provides a genetically encoded transmembrane scaffold for EV surface engineering. PTGFRN is enriched in certain human small EV preparations and was validated by Dooley and colleagues as a versatile scaffold for EV loading and surface display [27]. In a comparative scaffold screen, PTGFRN produced high levels of EV-associated GFP and generated a relatively uniform GFP-positive EV population compared with several conventional EV-localizing scaffolds, including tetraspanins and LAMP2b. These findings provided an experimental basis for using PTGFRN as a high-capacity EV scaffold. PTGFRN fusion proteins have since been used to display diverse biologically active proteins on EV surfaces, including cytokines, TNF superfamily ligands, and antibody-derived targeting domains [27]. This scaffold formed a core component of Codiak BioSciences’ clinical-stage EV platform. In exoIL-12, for example, IL-12 was displayed on the EV surface using PTGFRN to promote local immune activation after intratumoral administration while limiting systemic cytokine exposure [28]. Thus, PTGFRN represents a useful benchmark for high-density and relatively uniform EV surface display, particularly when robust presentation of large or multimeric proteins is required.

### 2.5. PDGFR-TM-Based Scaffold

The platelet-derived growth factor receptor transmembrane domain (PDGFR-TM) has been used as a genetically encoded membrane anchor for displaying scFv targeting domains on EVs. In the GEMINI platform, Stranford and colleagues fused an anti-CD2 single-chain variable fragment (scFv) to PDGFR-TM to generate T cell-targeted EVs [29]. This strategy was compared with lactadherin C1C2-mediated phosphatidylserine anchoring, highlighting how different EV display formats can affect ligand loading, display uniformity, and receptor-specific targeting. Optimization of the anti-CD2 scFv construct improved EV-associated surface display and markedly enhanced binding to CD2-positive Jurkat T cells compared with non-targeted EVs [29].

Notably, PDGFR-TM-based anti-CD2 display produced more receptor-specific T cell binding than C1C2-based display in this platform, despite the higher EV loading observed with C1C2-mediated anchoring. Because CD2 engagement can promote receptor internalization, anti-CD2-targeted EVs were efficiently taken up by T cells, a cell type that is otherwise relatively difficult to deliver cargo into [30]. These findings support PDGFR-TM display as a useful strategy when uniform, receptor-specific EV targeting and functional delivery to lymphocytes are required.

### 2.6. GPI-Anchored Proteins for EV Surface Display

Glycosylphosphatidylinositol (GPI)-anchored proteins are tethered to the exoplasmic leaflet of cellular membranes and can be incorporated into EV membranes, thereby presenting ligand-binding domains in an outward-facing orientation. Kooijmans and colleagues exploited this property to display anti-EGFR nanobodies on EV surfaces via GPI-anchor fusion, generating EVs that showed enhanced association with EGFR-expressing tumor cells in an EGFR-density-dependent manner [31]. GPI-anchored display offers a membrane geometry distinct from conventional transmembrane-domain fusions because the displayed domain is attached through a glycolipid anchor rather than a polypeptide transmembrane segment. However, the density and uniformity of GPI-anchored proteins on EVs may vary depending on producer cell type, GPI-anchor processing, membrane microdomain partitioning, and EV subtype composition. Therefore, GPI-anchored display requires optimization and validation for each therapeutic design.

### 2.7. Affinity-Based Modular Docking and Post-Isolation EV Functionalization

A complementary approach to genetically encoded EV surface display is modular functionalization through high-affinity molecular interactions. These strategies use peptide-protein, protein-protein, or biotin-streptavidin interactions to attach functional modules to EVs either after EV isolation or through engineered adaptor systems. By decoupling EV production from the final targeting, labeling, or cargo module, affinity-based docking enables rapid prototyping and flexible modification of a common EV preparation. One representative post-isolation strategy is based on CP05, a 12-amino acid peptide identified by Gao, Yin, and colleagues through phage display screening [32]. CP05 binds the second extracellular loop of CD63 with high affinity and specificity, allowing EVs from diverse sources to be decorated without genetic modification of producer cells. CP05-fused targeting ligands or therapeutic oligonucleotides can be incubated with purified or enriched EVs to generate functionalized vesicles, as shown by enhanced delivery of splice-correcting oligonucleotides in a Duchenne muscular dystrophy model. Other modular docking approaches include biotin-streptavidin systems using lactadherin as an EV membrane anchor and Fc/Protein A-based systems for cargo enrichment [33,34,35]. Streptavidin-lactadherin has been used both for EV labeling and biodistribution analysis, and for assembling tumor antigen-containing EV vaccines modified with biotinylated CpG DNA. Fc/Protein A-based docking has also been adapted for intraluminal cargo loading by recruiting functional proteins such as Cre recombinase or Cas9-based genome-editing components into engineered EVs.

Despite their flexibility, affinity-based docking systems require careful validation. CP05-based modification may depend on CD63 abundance, EV subtype composition, and accessibility of the CD63 extracellular loop. Biotin-streptavidin and Fc/Protein A systems may raise additional concerns, including complex stability in biological fluids, immunogenicity of adaptor components, unintended interactions with endogenous binding proteins, and cargo dissociation after administration. Thus, these systems are useful for rapid prototyping, EV labeling, biodistribution studies, vaccine assembly, and preclinical optimization, but therapeutic translation will require careful evaluation of cargo retention, biodistribution, immunogenicity, and repeat-dose safety.

An alternative chemical surface-engineering strategy that bypasses adaptor protein recruitment is bioorthogonal click chemistry. Producer-cell treatment with azide-modified sugars such as Ac4ManNAz introduces azide groups into surface glycans, which can be inherited by secreted EVs and subsequently conjugated with DBCO- or other strained-alkyne-functionalized ligands via copper-free SPAAC reactions. This enables covalent attachment of targeting ligands or imaging probes without genetic modification of the producer cells, as shown by Lim et al., who decorated EVs with PEGylated hyaluronic acid for CD44-mediated inflammatory targeting [36]. However, azide incorporation and conjugation efficiency must be optimized, and possible effects on EV biogenesis, surface composition, and uptake should be validated.

### 2.8. Comparative Considerations for Surface Engineering Scaffolds

The scaffolds described above differ in display capacity, uniformity across the EV population, immunogenicity risk, and compatibility with post-isolation modification, and these properties influence which platform is most suitable for a given therapeutic objective. Tetraspanin-based scaffolds offer broad compatibility and proven utility for small peptides or single-domain ligands but decorate only a fraction of the total EV pool, because CD9, CD63, and CD81 mark partially overlapping subsets [17]. Lamp2b enables N-terminal peptide display and supports brain-tropic targeting yet is prone to proteolytic cleavage unless protected by glycoengineering. MFG-E8/C1C2 anchoring decouples production from functionalization and is convenient for plug-and-play decoration, but its uniformity depends on phosphatidylserine exposure, which varies with EV source and isolation method. PTGFRN supports high-density, relatively uniform display of large or multimeric proteins, making it well-suited to surface-tethered cytokines and antibody-derived domains, although it has so far been developed mainly on a single producer-cell background. PDGFR-TM provides uniform receptor-specific display and has been used for lymphocyte-targeted scFv presentation. GPI anchoring offers a distinct membrane geometry but requires per-design optimization of microdomain partitioning. Affinity-based docking systems (CP05, biotin/streptavidin, Fc/Protein A) enable rapid prototyping but bring potential concerns over complex stability, adaptor immunogenicity, and cargo dissociation in vivo. In practice, scaffold choice should reflect the intended displayed molecule, the recipient cell type, the required dose, and the regulatory tolerance for genetic versus post-isolation modification, rather than display efficiency alone.

## 3. Cargo Loading Strategies

The therapeutic efficacy of engineered EVs depends not only on precise surface targeting but also on the efficient and selective incorporation of bioactive cargoes. Cargo loading strategies can be broadly organized according to when loading occurs and how cargoes are recruited into EVs. In terms of timing, cargoes can be loaded during EV biogenesis through producer-cell engineering or after EV isolation using physical, chemical, or affinity-based methods. Mechanistically, cargo incorporation may be actively driven by engineered protein-protein or protein-RNA interactions, or may rely on less specific processes such as cargo overexpression, membrane association, or endogenous cellular trafficking pathways.

Loading during EV biogenesis is attractive for therapeutic development because it minimizes direct manipulation of isolated EVs and may be more compatible with scalable manufacturing. However, this approach can be influenced by producer-cell biology, cargo toxicity, and potential changes in EV composition caused by genetic engineering or cargo overexpression. In contrast, post-isolation loading provides greater flexibility, particularly for small-molecule drugs, chemically modified nucleic acids, proteins, or personalized formulations. Nevertheless, physical or chemical manipulation after EV isolation can perturb membrane integrity, promote aggregation, or complicate interpretation of functional delivery (Figure 1).

### 3.1. RNA Loading via CD9-HuR: AU-Rich Element-Dependent Packaging

Li and colleagues developed an RNA-loading strategy based on fusion of the RNA-binding protein HuR, also known as ELAVL1, to the EV-enriched tetraspanin CD9 [13]. HuR recognizes AU-rich elements (AREs), which are commonly found in the 3′ untranslated regions of many mRNAs and regulate RNA stability and translation [37]. By tethering HuR to CD9, ARE-containing RNA cargoes can be recruited to the inner surface of EV membranes during EV biogenesis and enriched in secreted EVs. After uptake by recipient cells, these EV-delivered RNA cargoes retain functional activity and can induce gene-silencing effects. This strategy illustrates how RNA-binding proteins can be fused to EV-localizing scaffolds to promote selective RNA incorporation. A key limitation is that HuR recognizes a broad class of ARE-bearing sequences rather than a single defined synthetic tag [38]. Endogenous ARE-containing RNAs from producer cells may therefore be co-packaged with the intended therapeutic RNA or compete for HuR binding. Loading efficiency and cargo purity may depend on producer-cell type, HuR expression level, and the transcriptomic background of the donor cells.

### 3.2. Aptamer-Adapter Systems for Programmable RNA Loading

Aptamer-adapter systems provide a sequence-defined approach to EV RNA loading by using high-affinity interactions between engineered RNA motifs and cognate RNA-binding proteins. Representative tag-adapter pairs include MS2 coat protein/MS2 stem-loop, bacteriophage λ N peptide/boxB hairpin, and L7Ae/C/D box systems [39,40,41]. In EV-loading applications, the RNA-binding adapter is fused to an EV-localizing scaffold such as Lamp2b, CD63, CD9, or another EV-enriched membrane-associated protein, while one or more copies of the corresponding RNA tag are appended to the cargo RNA. This design enables selective recruitment of engineered RNA cargoes into EVs without relying on broad endogenous sequence classes such as AU-rich elements. The MS2/MCP system has been used in modular EV loading strategies, in which MCP is fused to EV-associated proteins and MS2 stem-loops are engineered into cargo RNAs to enhance EV enrichment [42]. Similarly, the compact L7Ae/C/D box pair can recruit C/D box-tagged mRNAs into EVs when L7Ae is fused to an EV-localizing scaffold such as CD63 [11]. Tandem aptamer arrays may further increase recruitment efficiency by providing multiple binding sites, although larger RNA tags can affect cargo RNA stability, translation, or release after delivery.

Overall, aptamer-adapter systems illustrate how synthetic RNA tags can convert EV cargo loading from a largely passive process into a programmable recruitment mechanism. However, loading efficiency can depend on adapter expression, scaffold topology, RNA tag copy number, cargo RNA size, and accessibility of the RNA motif. In addition, strong adapter-RNA interactions may retain cargo near the EV membrane and limit functional release in recipient cells, making cargo release and endosomal escape important considerations for therapeutic applications.

### 3.3. Sequence-Specific sgRNA Loading via Tetraspanin-dCas9

Catalytically inactive Cas9, or dCas9, fused to CD63 provides another strategy for RNA cargo recruitment. In this system, dCas9 is anchored to the inner surface of EV membranes through its fusion to CD63. When producer cells co-express CD63-dCas9 and an sgRNA, the sgRNA forms a ribonucleoprotein complex with the membrane-tethered dCas9 and is co-packaged into secreted EVs [43]. Unlike HuR-mediated loading, this approach is driven by the sequence-independent but guide-specific interaction between dCas9 and sgRNA, rather than by broad endogenous RNA classes such as AREs. Because guide identity is determined by sgRNA sequence, this strategy can in principle be adapted to different targets simply by changing the guide RNA. Similar RNP-loading logic could potentially be extended to catalytically active Cas9 or other genome-editing systems, although therapeutic application would require careful optimization of editing efficiency, cargo stoichiometry, intracellular release, and off-target activity [44,45,46]. In particular, EV-mediated genome-editing delivery remains challenging because functional editing requires not only efficient cargo loading but also cytosolic and nuclear access in recipient cells.

### 3.4. Photo-Controlled Cargo Loading and Release

Light-controlled systems provide temporal control over EV protein cargo loading or release. Two representative strategies are EXPLOR and MAPLEX, which regulate distinct steps of the delivery process. EXPLOR, or exosomes for protein loading via optically reversible interactions, uses blue-light-induced heterodimerization between CRY2 and CIBN [12]. In this system, CIBN is fused to an EV-localizing scaffold such as CD9, while CRY2 is fused to the protein cargo. Blue-light exposure recruits the cargo to EV-forming membranes during biogenesis, whereas removal of light allows dissociation of the interaction after EV production. This enables transient, user-controlled cargo loading and has been applied to protein cargoes such as fluorescent proteins, recombinases, and signaling regulators.

MAPLEX controls cargo release rather than recruitment [35]. In this system, a photocleavable linker is placed between the cargo and an EV-tethering domain. Violet-light irradiation cleaves the linker, separating the cargo from its EV anchor and promoting functional intracellular delivery after uptake by recipient cells. MAPLEX may therefore be useful for protein cargoes whose activity requires release from the EV scaffold and access to cytoplasmic or nuclear targets. Although these approaches provide precise temporal control, their requirement for light exposure makes them most practical in controlled ex vivo manufacturing or experimental settings.

### 3.5. Retroelement-Derived Capsid-Mediated RNA Packaging: Arc and PEG10/SEND

Some extracellular RNA-delivery systems use retroelement-derived capsid-like proteins rather than conventional EV luminal loading. The neuronal protein Arc self-assembles into retrovirus-like capsids that encapsulate RNA and mediate intercellular RNA transfer [47,48]. This finding suggests that retroelement-derived Gag-like proteins can be repurposed for extracellular RNA communication.

A more programmable example is SEND, or selective endogenous encapsidation for cellular delivery. SEND uses PEG10, a mammalian retrotransposon-derived Gag-like protein, to form virus-like particles that preferentially package transcripts containing PEG10 untranslated regions [49]. When combined with fusogenic proteins such as VSV-G or endogenous syncytins, PEG10-derived particles can deliver functional mRNAs, including Cre or genome-editing components, to recipient cells [50,51].

These systems are conceptually relevant to EV engineering because they demonstrate how extracellular particles can be programmed to package and deliver defined RNA cargoes. However, Arc-, PEG10-, and VLP-based systems should be distinguished from conventional EV cargo loading, because RNA is packaged within capsid-like or virus-like particles rather than freely incorporated into classical EV lumens. Thus, they are best considered EV-adjacent RNA delivery platforms that inform, but are mechanistically distinct from, standard EV-loading strategies. In nomenclature terms, Arc-, PEG10-, and VLP-based particles are most accurately described as engineered virus-like or capsid-like RNA-delivery particles that share producer-cell biology and extracellular trajectories with EVs, rather than as engineered EVs per se.

### 3.6. Small-Molecule Drug Loading

In addition to nucleic acids and proteins, EVs have been widely investigated as carriers for small-molecule drugs, particularly chemotherapeutic and anti-inflammatory agents. Small-molecule loading can be achieved by passive incubation, electroporation, sonication, freeze–thaw cycling, saponin-assisted permeabilization, or producer-cell preloading [52]. The optimal method depends on the physicochemical properties of the drug, including hydrophobicity, charge, molecular weight, membrane permeability, and stability.

Passive incubation is technically simple and is especially suitable for hydrophobic drugs that partition into EV membranes, such as curcumin or paclitaxel [53]. Electroporation, sonication, freeze–thaw cycling, and saponin-assisted permeabilization can enhance loading of less membrane-permeable compounds by transiently disrupting EV membranes [20,54,55]. However, these approaches may induce vesicle aggregation, membrane damage, cargo precipitation, or changes in EV surface proteins and biological activity. Producer-cell preloading provides an alternative strategy in which donor cells are exposed to a drug that becomes incorporated into secreted EVs through endogenous cellular pathways [56]. This avoids direct manipulation of isolated EVs but may alter producer-cell physiology and EV composition. Small-molecule EV loading is among the more clinically developed areas of EV-based therapeutic research. Nevertheless, because loading procedures can alter EV integrity or generate non-EV drug-containing particles, rigorous assessment of drug-to-EV ratio, free-drug contamination, vesicle recovery, structural integrity, and functional activity is essential.

### 3.7. Cellular Nanoporation for High-Yield mRNA EV Production

Yang and colleagues introduced cellular nanoporation as a producer-cell-based strategy to enhance EV production and enrich therapeutic mRNAs in EVs [57]. In this approach, producer cells are cultured on a nanochannel-patterned silicon chip and exposed to brief, localized electrical pulses, which promote plasmid DNA entry into cells while stimulating EV release. The resulting EVs contain mRNAs transcribed within producer cells and incorporated during endogenous EV biogenesis, thereby avoiding direct electroporation of isolated vesicles and the associated risks of vesicle disruption, RNA aggregation, and loading artifacts. Yang and colleagues reported markedly increased EV yield and exosomal mRNA levels compared with conventional bulk electroporation or lipofection-based approaches. In an orthotopic glioma model, PTEN mRNA delivered by nanoporation-derived EVs restored tumor-suppressor activity, inhibited tumor growth, and improved survival. Thus, cellular nanoporation represents a hybrid strategy that combines producer-cell transfection, stimulation of EV biogenesis, and endogenous mRNA packaging. Remaining challenges include scaling the chip-based system for GMP-compatible manufacturing and ensuring reproducible EV composition and potency.

### 3.8. Comparative Considerations and Technical Caveats in EV Cargo Loading

Comparative studies of EV cargo loading methods have highlighted technical caveats that can complicate the interpretation of EV-mediated delivery. These issues are especially important for post-isolation loading, where physical or chemical manipulation may alter EV integrity or generate non-EV cargo-containing particles. For example, electroporation can induce RNA aggregation, vesicle disruption, and particle aggregation, and cargo-containing aggregates may co-purify with EVs [58,59]. Similarly, cationic lipid reagents can form lipoplexes that deliver cargo independently of EVs, making no-EV and reagent-only controls essential [60,61]. Producer-cell-based strategies, including RNA-binding protein recruitment, affinity-tag systems, optogenetic recruitment, and cellular nanoporation, avoid direct manipulation of isolated EVs but are not artifact-free. Producer-cell engineering, cargo overexpression, altered EV composition, and batch-to-batch variability can all influence the final EV product. To make these requirements concrete, EV cargo-loading studies should report, at a minimum: (i) cargo amount per particle, together with a no-EV or reagent-only control to exclude lipoplex- or aggregate-mediated delivery; (ii) cargo protection assays demonstrating nuclease or protease resistance that is lost on detergent treatment, confirming intra-vesicular localization; (iii) density-gradient or size-exclusion co-fractionation showing that the cargo co-migrates with bona fide EV markers; (iv) single-particle analysis (e.g., single-particle interferometric reflectance imaging, super-resolution microscopy, or single-EV flow) when co-residence of multiple engineered modules on the same vesicle is claimed; and (v) recipient-cell functional readouts that distinguish productive intracellular delivery from passive uptake or surface binding, for which reporter-based assays of EV-mediated membrane fusion and cytosolic cargo delivery provide quantitative benchmarks [62]. These controls need not all be performed in every study, but their absence should be explicitly acknowledged when interpreting potency or therapeutic claims [1].

## 4. Engineered EVs in Cancer Immunotherapy

Cancer immunotherapy has been transformed by immune checkpoint blockade, adoptive cell therapy, and therapeutic vaccination, yet major challenges remain, including limited penetration into solid tumors, systemic toxicity, immunosuppressive tumor microenvironments, and the manufacturing complexity of patient-specific cellular products. Engineered EVs have therefore attracted increasing attention as programmable, cell-free therapeutic particles capable of delivering defined immune signals, cytotoxic payloads, nucleic acids, and checkpoint-modulating molecules [63]. A biological rationale for this approach comes from tumor-derived EVs, which naturally modulate anti-tumor immunity, remodel the tumor microenvironment, and promote metastatic progression [3]. By carrying immunosuppressive ligands, TGF-β, and regulatory RNA cargoes, tumor-derived EVs can impair cytotoxic T cells, dendritic cells, NK cells, and other immune effector populations. Therapeutic EV engineering can therefore be viewed as an attempt to redirect this biology: engineered EVs are designed to replace suppressive tumor-derived signals with controlled activating, cytotoxic, antigenic, or checkpoint-modulating signals.

### 4.1. EV Vaccines and Antigen Presentation

Dendritic cell-derived EVs were among the earliest EV-based cancer vaccine platforms [64]. These vesicles carry MHC–peptide complexes, co-stimulatory molecules, and immune-activating proteins, allowing them to initiate or amplify T cell responses without administration of live dendritic cells [65,66]. Early preclinical studies and clinical trials in melanoma and non-small cell lung cancer showed that dendritic cell-derived EVs can be produced safely and induce measurable immunological activity, although clinical efficacy has remained modest [67,68,69]. These studies nevertheless established an important precedent for cell-free EV-based cancer vaccination.

Subsequent engineering strategies have aimed to improve EV vaccine potency by combining antigen delivery with defined immunostimulatory signals. EVs can be loaded or decorated with tumor antigens, neoantigen peptides, adjuvants such as CpG oligonucleotides, or alarmin-like molecules that promote dendritic cell maturation [34,70,71,72]. In this context, EVs act as scaffolds that co-deliver antigen and immune stimulation to antigen-presenting cells, thereby enhancing cross-presentation and cytotoxic T cell priming.

A more synthetic approach is to engineer antigen-presenting EVs (AP-EVs) with defined peptide–MHC complexes, selected co-stimulatory ligands, and cytokine modules [10,73,74,75]. Such EVs function as programmable immune synapse mimics, enabling control over antigen specificity, co-stimulation, cytokine support, and signal density. Because simple MHC overexpression may not ensure efficient incorporation or optimal surface display on EVs, fusion of peptide–MHC complexes to EV-enriched scaffold proteins may help enhance EV association and improve control over antigen display.

### 4.2. Immune Cell-Derived EVs as Cytotoxic and Immunomodulatory Effectors

EVs released by immune effector cells can retain key functional features of their parental cells. NK cell-derived EVs contain cytotoxic molecules such as perforin and granzyme B and may carry receptors involved in tumor recognition, enabling MHC-unrestricted tumor cell killing [76]. Their antitumor activity can be further enhanced by engineering tumor-targeting receptors or loading therapeutic cargoes such as pro-apoptotic molecules, cytokines, or siRNAs [77].

T cell- and CAR T cell-derived EVs represent another class of immune effector EVs. Activated CD8^+^ T cell-derived EVs can remodel the tumor stroma, including cancer-associated fibroblasts and mesenchymal stromal components, thereby extending their function beyond direct tumor cell killing [78]. CAR T cell-derived EVs are particularly attractive because they can display CAR molecules while carrying cytotoxic proteins such as perforin and granzyme B [79]. These EVs retain antigen-directed cytotoxicity and may reduce risks associated with live CAR T cell infusion, including excessive cytokine release. Multifunctional CAR T EVs may further combine CAR-mediated recognition with regulatory RNA cargoes to attack tumors through complementary mechanisms [80].

M1-like macrophage-derived EVs provide a distinct therapeutic logic. Rather than acting primarily as direct cytotoxic particles, they can reprogram the tumor microenvironment by transferring inflammatory cargoes that shift tumor-associated macrophages toward states that support antigen presentation and T cell activity [81,82]. This strategy may synergize with checkpoint blockade by converting an immunosuppressive tumor microenvironment into one that is more permissive for adaptive immune responses.

### 4.3. RNA-Loaded EVs for Oncogene Silencing and Cytokine Expression

EVs can also serve as carriers for RNA therapeutics in cancer. A well-studied example is the delivery of KRAS mutant-specific siRNA or shRNA to pancreatic ductal adenocarcinoma, where engineered exosomes suppress KRAS-driven signaling and reduce tumor growth in preclinical models [83]. This strategy has also been evaluated in early clinical testing with engineered exosomes carrying KRASG12D-specific siRNA [84]. Their activity may be supported by the macropinocytic nature of KRAS-mutant cancer cells and by EV-mediated protection of RNA cargoes. EVs can also deliver immunostimulatory RNAs that reshape the tumor microenvironment. Inhalable EVs carrying IL-12 mRNA illustrate this concept in lung cancer models, where local cytokine expression can activate NK cells and cytotoxic T cells while potentially limiting systemic toxicity [85]. Thus, EV RNA therapeutics can be designed either to suppress tumor-intrinsic pathways or to reprogram local immunity through transient expression of immunostimulatory factors.

### 4.4. EVs for Immune Checkpoint Modulation

Because tumor-derived EVs can disseminate checkpoint ligands such as PD-L1, engineered EVs have been developed to block checkpoint pathways or exploit checkpoint molecules as tumor-targeting handles [86,87]. EVs can be engineered to display checkpoint-binding proteins or antibodies, allowing checkpoint blockade to be combined with local delivery of therapeutic cargoes [88,89]. In tumors with high PD-L1 expression, EVs can also be engineered to target PD-L1-positive cancer cells while delivering inhibitory RNA cargoes such as STAT3 siRNA [90]. In this case, surface targeting and intraluminal gene silencing are combined to disrupt both immune evasion and tumor-intrinsic survival pathways. Beyond the PD-1/PD-L1 axis, EVs have also been designed to modulate phagocytic checkpoints. For example, SIRPα-displaying EVs can bind tumor CD47 and enhance macrophage phagocytosis [91]. Together, these studies show that engineered EVs can modulate checkpoint biology in several ways. They can counteract suppressive tumor EV mechanisms, block inhibitory receptor-ligand interactions, enhance phagocytosis, or deliver RNA cargoes that suppress checkpoint-associated or tumor-intrinsic survival pathways. Thus, checkpoint-modulating EVs expand the role of EV therapeutics beyond antigen delivery and cytotoxicity into the active remodeling of tumor immune suppression.

### 4.5. Design Principles for Cancer Immunotherapy EVs

Across these platforms, engineered EVs in cancer immunotherapy can be organized around several design principles. First, EVs can function as antigen-presenting particles that display peptide–MHC complexes, tumor antigens or neoantigen-derived peptides, and co-stimulatory ligands. Second, they can act as effector particles carrying cytotoxic proteins, chimeric or decoy immune receptors, cytokines, or regulatory RNAs. Third, EVs can reprogram the tumor microenvironment by altering macrophage polarization, dendritic cell activation, stromal cell survival, or local cytokine balance. Fourth, they can modulate immune checkpoint pathways by blocking suppressive interactions or targeting checkpoint-positive tumor cells.

Next-generation EV platforms are likely to combine several of these functions within a single particle. For example, EV vaccines may co-deliver antigen and adjuvant, CAR T cell-derived EVs may combine receptor-mediated targeting with cytotoxic cargo, and RNA-loaded EVs may simultaneously target tumor cells and reshape immune signaling. This modularity is a central advantage of EV engineering: unlike natural EVs, whose activity largely reflects the state of the parental cell, engineered EVs can be programmed through defined surface ligands, cargoes, and production strategies.

Clinical translation will require careful control of manufacturing, batch consistency, cargo stoichiometry, biodistribution, tumor penetration, immune clearance, and safety after repeated administration [1]. Future studies should also distinguish functional delivery from simple cellular uptake and define how surface ligands, cargo density, and recipient-cell specificity determine therapeutic efficacy.

## 5. Engineered EVs in Immune Modulation and Tolerance

In addition to their immunostimulatory applications, EVs are natural mediators of immune suppression, inflammation resolution, and peripheral tolerance [92]. EVs released by mesenchymal stromal cells, Tregs, tolerogenic dendritic cells, macrophages, and tissue-resident cells can dampen effector immune responses, promote regulatory cell populations, and reshape inflammatory tissue environments [93,94,95,96]. This endogenous tolerogenic capacity provides a foundation for engineering EVs as therapeutic tools for immune regulation. Through modification of surface molecules, targeting ligands, antigen-presenting complexes, checkpoint molecules, cytokines, enzymes, or RNA cargoes, broad anti-inflammatory EV activity may be converted into more potent, precise, disease-relevant, and potentially antigen-specific immune regulation (Figure 2c).

### 5.1. MSC-Derived EVs as Broad Immunomodulatory Therapeutics

Among tolerogenic and anti-inflammatory EV sources, mesenchymal stromal cell-derived EVs are the most widely studied [97]. Unlike antigen-specific tolerogenic EVs, MSC-derived EVs generally act through broad, context-dependent immunomodulatory mechanisms rather than defined antigen recognition. They carry immunoregulatory proteins, lipids, metabolites, and non-coding RNAs that can affect dendritic cells, macrophages, T cells, B cells, and NK cells [98].

Preclinical studies have demonstrated MSC-EV activity in multiple autoimmune and inflammatory disease models, including inflammatory arthritis, autoimmune neuroinflammation, type 1 diabetes, uveitis, graft-versus-host disease, and acute inflammatory injury [99,100,101,102,103]. These findings support the concept that MSC-EVs can attenuate inflammatory immune circuits across distinct disease contexts. Early clinical experience, particularly in graft-versus-host disease and severe inflammatory disorders, has suggested the feasibility of MSC-EV administration as a potential cell-free alternative to MSC therapy, although clinical evidence remains limited [104].

MSC-EVs can also be engineered to improve their therapeutic utility. Surface modification may enhance targeting to inflamed tissues or specific immune cell populations [105], metabolic glycoengineering can enable chemical conjugation of ligands or therapeutic molecules [36], and drug loading can combine intrinsic MSC-EV immunomodulation with defined anti-inflammatory agents such as rapamycin [106]. Thus, MSC-EVs provide a practical entry point for tolerogenic EV therapy, combining endogenous immunomodulatory activity with opportunities for disease-directed engineering. However, major translational challenges remain, including donor and tissue-source variability, batch heterogeneity, lack of standardized potency assays, incomplete mechanism-of-action definition, and scalable GMP-compatible manufacturing.

### 5.2. Natural Tolerogenic EV Pathways

In parallel with MSC EV studies, work on Treg, tolerogenic dendritic cell, and apoptotic cell-derived EVs has clarified how EVs naturally transmit immune regulatory signals. Tolerogenic dendritic cell EVs can present MHC–peptide complexes together with regulatory signals, thereby modulating antigen-specific T cell responses without necessarily inducing full effector activation [95]. Apoptotic cell-derived EVs and apoptotic bodies can promote immune quiescence through efferocytosis, tolerogenic antigen presentation, and anti-inflammatory cytokine production [107].

Treg cell-derived EVs provide one of the clearest examples of naturally suppressive EVs. Treg EVs can express CD73, enabling adenosine mediated immune regulation, and can transfer regulatory microRNAs that suppress inflammatory T helper cell responses [108,109]. They can also alter dendritic cell function, indicating that Treg EVs suppress immunity both directly and indirectly through antigen-presenting cells.

These natural tolerogenic EV pathways are conceptually important because they show that EVs can suppress immunity through combinations of surface enzymes, regulatory proteins, and RNA cargoes. However, naturally produced tolerogenic EVs are heterogeneous, often lack defined antigen specificity, and may show variable biodistribution. Engineering strategies therefore aim to preserve their suppressive activity while improving targeting, reproducibility, potency, and antigen specificity.

### 5.3. Engineered EVs for Autoimmune and Inflammatory Diseases

Although MSC EVs and other natural tolerogenic EVs provide broad immune suppression, many autoimmune and inflammatory diseases require more precise control of tissue localization, immune pathway engagement, or antigen specificity. Engineered EVs are therefore being developed to target inflamed tissues, co-deliver immunomodulatory cargoes, and selectively suppress pathogenic immune pathways.

Inflammatory bowel disease provides one example. Hybrid vesicles combining inflammatory tissue-homing membranes, Treg-derived immunosuppressive cargo, and pathway-specific biologics such as anti-IL-23 antibodies have been designed to accumulate in inflamed gut mucosa and suppress IL-23/Th17-driven inflammation [110]. Autoimmune neuroinflammation provides another example. Anti-inflammatory macrophage-derived EVs decorated with myelin-associated peptide antigens have been developed to combine an immunoregulatory vesicle background with disease-relevant antigen specificity [111]. This design aims to reduce pathogenic autoreactivity while limiting broad systemic immunosuppression.

More generally, engineered EVs can be modified to target inflamed endothelium, activated macrophages, autoreactive lymphocytes, or disease specific stromal niches. Their cargoes can include anti-inflammatory miRNAs, siRNAs, cytokines, checkpoint ligands, enzymes, or small molecules. The goal is not simply to increase EV uptake, but to coordinate biodistribution, immune cell targeting, and suppressive signaling in a disease-relevant manner.

### 5.4. Tolerogenic Antigen-Presenting EVs for Antigen-Specific Immune Regulation

Broad immunosuppression can impair protective immunity against pathogens and tumors. Therefore, antigen-specific tolerance, which selectively suppresses immune responses against defined self-antigens or alloantigens, represents a more precise therapeutic strategy. Tolerogenic antigen-presenting EVs aim to achieve such selective immune regulation by combining antigen recognition with inhibitory or regulatory co-signals. In this design, defined MHC–peptide complexes are displayed on EVs together with tolerogenic signals such as PD-L1 or surface-tethered TGF-β [75,112]. Antigen recognition through the T cell receptor provides specificity, while co-inhibitory and regulatory signals are expected to promote the induction, stabilization, or expansion of antigen-specific Foxp3-positive Tregs. Thus, the basic architecture of antigen-presenting EVs used for immune activation can be repurposed for tolerance by altering the co-signal configuration. Such EVs could be applied to diseases in which relevant antigens or alloantigens are defined, including type 1 diabetes, multiple sclerosis, allergy, and transplantation. Key issues to optimize include antigen density, co-signal composition, EV dose, target cell specificity, durability of tolerance, and safety. Nevertheless, tolerogenic antigen-presenting EVs represent an important approach beyond broad EV-mediated immunosuppression toward programmable antigen-specific immune tolerance.

### 5.5. Risks of EV-Mediated Immune Suppression

Broad or insufficiently targeted immunosuppressive EVs raise safety considerations that warrant explicit attention as the field moves toward clinical use. Systemic delivery of EVs decorated with PD-L1, TGF-β, or other tolerogenic ligands could, in principle, dampen protective immunity against tumors and pathogens, reactivate latent infections, or impair vaccine responses if biodistribution is not tightly controlled. Antigen-non-specific MSC-EVs raise distinct concerns regarding chronic low-grade immunosuppression on repeat dosing, particularly in patients with concurrent malignancy or infection risk [113]. Engineering strategies that combine antigen-specific recognition with controlled tissue or cell-type targeting are therefore not only a route to higher potency but also a key safety strategy. Future tolerogenic EV programs should include readouts for off-target immune suppression, including effects on anti-tumor immunity, response to vaccination, and reactivation of latent viral infections.

## 6. Clinical Translation of Engineered EV Therapeutics

The clinical translation of engineered EV therapeutics remains at an early stage. A limited number of EV-based products have entered clinical testing, providing initial evidence of feasibility, acceptable short-term safety, and pharmacodynamic or immunological activity. However, definitive clinical efficacy has not yet been established for most programs, and key challenges remain, including standardized potency assays, scalable manufacturing, reproducible batch characterization, quantitative dose metrics, and clear regulatory pathways. The current landscape should therefore be viewed as an emerging proof-of-concept field rather than a mature therapeutic modality. Representative clinical and translational programs have used diverse EV sources and delivery routes, including RNA-loaded EVs, rationally engineered EV platforms, and selected cargo-modified or overexpression-based EV products (Table 2), while unmodified MSC-EVs and antigen-loaded dendritic cell EVs are discussed separately in the text.

Early dendritic cell EV vaccine studies showed that EVs can be prepared from autologous immune cells and administered safely, with detectable immunological activity in some patients [120]. However, individualized dendritic cell culture is labor-intensive and difficult to standardize, limiting broad clinical scalability. RNA-loaded EV programs, such as KRAS-targeted iExosomes for pancreatic cancer, represent an important next step by evaluating EVs as carriers of therapeutic nucleic acids, although early-phase safety and target-engagement signals should not be interpreted as definitive efficacy [84].

Rationally engineered EV platforms, including exoSTING and exoIL-12, further demonstrate that EVs can be designed to engage defined immune pathways in clinical settings [28,114]. At the same time, the pausing or deprioritization of some programs highlights that clinical translation depends not only on scientific feasibility, but also on manufacturing robustness, financing, strategic prioritization, and organizational sustainability. MSC-derived EV studies in GVHD and severe inflammatory conditions provide a separate precedent for cell-free immunomodulation, but remain limited by small study size, incomplete mechanism-of-action definition, and the need for robust potency assays [121,122,123].

Overall, clinical experience to date supports cautious optimism. EV therapeutics can be manufactured, administered by multiple routes, and evaluated for biological activity in humans. The next phase of development will require tighter control of product heterogeneity, cargo loading, biodistribution, long-term safety, and comparability after manufacturing changes. Progress in these areas will determine whether engineered EVs can advance from promising translational platforms to clinically reliable therapeutic products.

Several recurring obstacles help explain why EV platforms have moved more slowly from preclinical proof-of-concept to clinical efficacy than initial reports suggested. After systemic administration, unmodified EVs accumulate predominantly in liver, spleen, and lungs, with limited penetration into solid tumors or central nervous system tissue. Quantitative human biodistribution data also remain scarce and are not always congruent with murine models. Even when EVs reach the intended cell type, productive cargo delivery requires endosomal escape, which remains incompletely characterized for engineered EVs and may limit protein or RNA cargo function. Uneven biodistribution and inefficient endosomal escape are therefore key mechanistic reasons why pharmacodynamic activity in early-phase trials may outpace clear efficacy signals, and both should be measured directly in next-generation programs.

When engineered EVs are evaluated against competing modalities such as lipid nanoparticles (LNPs), adeno-associated viral vectors, virus-like particles, antibody-drug conjugates, recombinant cytokines, synthetic nanoparticles, and live cellular therapies, several relative weaknesses become apparent. Compared with LNPs, engineered EVs currently have less defined composition, lower per-batch potency, and more challenging analytical characterization. Compared with viral vectors and VLPs, they generally achieve lower intracellular delivery efficiency, and compared with recombinant cytokines or antibody-drug conjugates, they require more complex manufacturing and less standardized potency assays. Their complementary advantages, including programmable multi-module composition, intrinsic cell-derived biocompatibility, and the ability to combine surface targeting with luminal cargo in a single particle, are therefore best leveraged where these features are decisive. Examples include local intratumoral immune modulation, antigen-specific tolerance induction, and co-delivery of multiple signals to defined immune populations, rather than use as a generic substitute for established delivery platforms.

Clinical translation also depends on factors beyond molecular design. The pausing or discontinuation of the Codiak BioSciences exosome programs (exoSTING, exoIL-12, exoASO-STAT6) for financial and organizational rather than safety reasons (Table 2) illustrates the field’s sensitivity to commercial sustainability, capital availability, and shifting therapeutic priorities. The choice of “personalized” engineering configuration, including donor cell source, scaffold, cargo, route, and combination partner, is therefore unlikely to follow a universal recipe and must be tailored to indication, target cell, regulatory pathway, and manufacturing scale. Realistically, broad clinical impact of engineered EVs in immunotherapy and tolerance induction is likely to unfold over a five- to fifteen-year horizon, with earlier indication-restricted approvals plausible for locally administered intratumoral platforms or orphan-disease applications where existing alternatives are limited.

## 7. Conclusions and Future Perspectives

Engineered extracellular vesicles are emerging as programmable immune interfaces rather than simple delivery vehicles. By integrating surface display, targeting, cargo loading, and defined immune signals, EVs can be designed to direct immune responses in a context-dependent manner. In cancer immunotherapy, engineered EVs may enhance antigen presentation, deliver therapeutic cargoes, remodel the tumor microenvironment, or modulate checkpoint pathways. In immune tolerance, the same design principles can be redirected toward anti-inflammatory signaling, regulatory cell induction, and antigen-specific immune suppression. For clinical translation, key challenges remain, including EV heterogeneity, reproducible manufacturing, cargo stoichiometry, biodistribution, potency assays, and long-term safety. Addressing these issues will be essential for transforming engineered EVs from promising experimental platforms into reliable therapeutic products for precision immune modulation.

Looking forward, the most realistic translational expectation is incremental rather than universal: the choice among scaffolds, producer cells, cargo strategies, and routes will need to be tailored to each indication, target cell, and regulatory context, and the most impactful first approvals are likely to emerge from locally administered or orphan-indication settings on a five- to fifteen-year horizon. Within this framing, engineered EVs offer not a single dominant platform but a flexible toolkit for precision immune modulation that complements, rather than replaces, established delivery modalities.

## Figures and Tables

**Table 1:** Modular design logic of engineered EVs as programmable immune interfaces.

Module	Design Question	Representative Engineering Approaches	Intended Immunological Outcome
**Targeting (address)**	Which recipient cell or tissue should the EV reach?	scFvs/nanobodies/peptides using EV scaffold proteins or affinity-based post-isolation attachment strategies (§2, §4.2)	Selective enrichment of EV interactions with defined target cells or tissues
**Surface signal (instruction)**	What immune instruction should be delivered at the EV-cell interface?	pMHC class I/II; co-stimulatory or TNF-family ligands such as CD80 and CD40L; cytokine signals such as IL-2; tolerogenic signals such as TGF-β or PD-L1 (§2.4, §4.1, §5.4)	Activation, inhibition, co-stimulation, cytokine support, or tolerance induction
**Cargo (payload)**	What intracellular process should change after EV uptake?	RNAs, proteins, genome/epigenome regulators, small molecules, or cytotoxic proteins (§3, §4.2–4.3)	Modulates recipient-cell function through cytotoxic, inflammatory, tolerogenic, or gene-regulatory effects.

**Note.** The modules are conceptual and not mutually exclusive; a single engineered molecule may contribute to both targeting and signaling depending on the receptor and recipient cell. Two requirements apply across all three modules: targeting should be validated separately from downstream signaling, and cargo activity should reflect productive intracellular delivery rather than mere cellular uptake.

**Table 2:** Selected engineered or cargo-modified extracellular vesicle therapeutics evaluated in clinical trials.

EV (Developer)	Source	Engineering	Payload/Signal	Indication; Route	Phase; Trial ID	Status and Key Findings	Ref.
**iExoKras^G12D^** MD Anderson	Bone-marrow MSCs	Cargo loading (siRNA electroporation)	Luminal KRAS^G12D^ siRNA	Metastatic PDAC (KRAS^G12D^); IV (± ipilimumab)	Phase I NCT03608631	Phase I Completed; no DLT; ↑ intratumoral CD8^+^ T cells.	[84]
**exoIL-12 (CDK-003)** Codiak	HEK293	Surface display (PTGFRN)	IL-12 on EV surface	Cutaneous T-cell lymphoma; intratumoral	Phase 1 NCT04592847/NCT05156229	Well tolerated; 1 PR observed. Discontinued following Codiak’s bankruptcy filing.	[28]
**exoSTING (CDK-002)** Codiak	HEK293 (PTGFRN)	Luminal loading (PTGFRN EVs)	STING agonist (cyclic dinucleotide)	Injectable solid tumors (HNSCC, TNBC, ATC, cSCC); intratumoral	Phase 1/2 NCT04592484	Well tolerated; tumor retention observed. Discontinued following Codiak’s bankruptcy filing.	[114]
**Drug-packaging tumor MPs** Huazhong Univ.	Tumor cell-derived microparticles (MPs)	Drug packaging (chemotherapeutic loading)	Methotrexate; cisplatin	MPE/ascites; biliary obstruction in cholangiocarcinoma; intracavitary/intrabiliary	Phase II NCT01854866/NCT02657460	Improved pleural-effusion response in a randomized study; relief of biliary obstruction in ~25%.	[115,116]
**ILB-202 (Exo-SR)** ILIAS Biologics	HEK293	Optogenetic protein loading (EXPLOR)	srIκB (NF-κB inhibitor)	CSA-AKI/inflammation; IV (healthy volunteers)	Phase 1 NCT05843799	Completed; safe, no immunogenicity (JEV 2025).	[117]
**EXO-CD24** Ichilov/OBCTCD24	HEK293 (CD24-overexpressing)	CD24 overexpression (surface enrichment)	CD24 (Siglec-10; ↓ cytokine storm)	COVID-19; ARDS; inhaled	Phase 1/2 NCT04747574/NCT04902183	Phase 1 favorable; Phase 2 in Greece.	[118]
**LDLR mRNA exosomes (ENDFH)** First-in-human, China	Bone-marrow MSCs	Producer-cell cargo loading (Ldlr vector)	LDLR mRNA	Homozygous familial hypercholesterolemia; IV	Phase I NCT05043181	First-in-human; results pending.	[119]

**Note.** Limited to EVs intentionally engineered by surface display, overexpression, or defined cargo loading; unmodified or preconditioned MSC/stem cell EVs and antigen-loaded dendritic cell EVs (Dex) are discussed in the text. Tumor-cell microparticles (MPs) are larger ectosome-type EVs. Several programs were paused for non-safety reasons; clinical feasibility and short-term safety are more firmly established than definitive efficacy at this stage. Trial information was last verified on 17 June 2026; readers are encouraged to consult ClinicalTrials.gov and equivalent registries for the most current status. Abbreviations: ATC, anaplastic thyroid carcinoma; CSA-AKI, cardiac surgery-associated acute kidney injury; cSCC, cutaneous SCC; DLT, dose-limiting toxicity; HNSCC, head and neck SCC; IV, intravenous; MP, microparticle; MSC, mesenchymal stromal cell; PDAC, pancreatic ductal adenocarcinoma; PR, partial response; PTGFRN, prostaglandin F2 receptor negative regulator; STING, stimulator of interferon genes; TNBC, triple-negative breast cancer. ↑, increased. ↓, decreased.

## Data Availability

Not applicable.
